# Mirror-enhanced scanning light-field microscopy for long-term high-speed 3D imaging with isotropic resolution

**DOI:** 10.1038/s41377-021-00665-9

**Published:** 2021-11-04

**Authors:** Bo Xiong, Tianyi Zhu, Yuhan Xiang, Xiaopeng Li, Jinqiang Yu, Zheng Jiang, Yihan Niu, Dong Jiang, Xu Zhang, Lu Fang, Jiamin Wu, Qionghai Dai

**Affiliations:** 1 Department of Automation, Tsinghua University, Beijing, 100084 China; 2 Institute for Brain and Cognitive Sciences, Tsinghua University, Beijing, 100084 China; 3 Beijing Laboratory of Brain and Cognitive Intelligence, Beijing Municipal Education Commission, Beijing, 100084 China; 4 State Key Laboratory of Membrane Biology, Tsinghua University-Peking University Joint Centre for Life Sciences, Beijing Frontier Research Center for Biological Structure, School of Life Sciences, Tsinghua University, Beijing, 100084 China; 5 Beijing Institute of Collaborative Innovation, Beijing, 100094 China; 6 Department of Electronic Engineering, Tsinghua University, Beijing, 100084 China

**Keywords:** Microscopy, Biophotonics

## Abstract

Various biological behaviors can only be observed in 3D at high speed over the long term with low phototoxicity. Light-field microscopy (LFM) provides an elegant compact solution to record 3D information in a tomographic manner simultaneously, which can facilitate high photon efficiency. However, LFM still suffers from the missing-cone problem, leading to degraded axial resolution and ringing effects after deconvolution. Here, we propose a mirror-enhanced scanning LFM (MiSLFM) to achieve long-term high-speed 3D imaging at super-resolved axial resolution with a single objective, by fully exploiting the extended depth of field of LFM with a tilted mirror placed below samples. To establish the unique capabilities of MiSLFM, we performed extensive experiments, we observed various organelle interactions and intercellular interactions in different types of photosensitive cells under extremely low light conditions. Moreover, we demonstrated that superior axial resolution facilitates more robust blood cell tracking in zebrafish larvae at high speed.

## Abstract (web summary)

Mirror-enhanced scanning light-field microscopy (MiSLFM) achieves long-term high-speed 3D imaging at near-isotropic subcellular resolution with a single objective, facilitating native observations of various intracellular and intercellular interactions.

## Graphical abstract

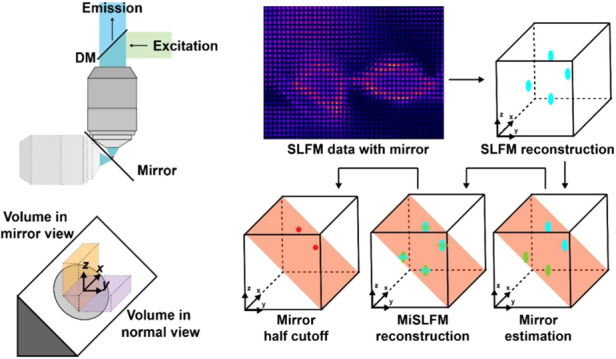

## Introduction

There is a complicated world in every cell with various intracellular and intercellular interactions of different organelles occurring in 3D at high speed, which is the basis of different physiological processes. However, current microscopy techniques usually image a certain plane at one time, with three-dimensional (3D) imaging obtained through movements of the focal plane relative to the specimen, such as confocal^1,2^, structured illumination^3–6^, and light-sheet microscopy^7–9^. To address this problem, a few emerging imaging techniques^10–15^ aimed at simultaneous 3D imaging have been developed recently. Among them, light-field microscopy (LFM)^16–18^ provides an elegant compact solution by capturing the excited volume simultaneously in a tomographic manner. The extended depth of field (DOF) of LFM keeps the light focused across a large range along different angles, facilitating high photon efficiency for 3D imaging. In this case, high-speed 3D fluorescence imaging can be obtained with extremely low light excitation, which is essential to observe native biological behaviors over the long term^19,20^. By incorporating high-speed drifting of the image plane^21,22^ in a scanning LFM (sLFM), the resolution can be further increased up to the diffraction limit of the entire objective numerical aperture (NA). However, as a wide-field collection method, LFM and sLFM still suffer from the common missing-cone problem, leading to much lower resolution in the axial domain than in the lateral domain^23,24^. Such axial resolution degradation poses a great challenge for accurate characterizations of organelle interactions in 3D, resulting in the failure of various quantitative analyses^25^.

By introducing additional illumination, both light-sheet microscopy^26^ and structured illumination microscopy^23^ can achieve axial super resolution at the cost of 3D imaging speed and system compactness. 4-pi microscopy^27,28^ uses two opposite objectives for coherent detection of fluorescence and improves both the resolution and SNR, while the sidelobe effect and complicated imaging setup restrict its broad applications in cell biology. By placing a mirror below the samples, the system can be greatly simplified^29,30^, but the shallow DOF prevents its application in high-speed 3D imaging. Multiview LFM with volumetric light-sheet illumination^31^ has thus been proposed to achieve high-speed 3D imaging at the isotropic resolution, enabling robust and accurate cell tracking in the beating heart of medaka. However, the resolution is not sufficient for subcellular structures, and a design with multiple objectives makes it hard to perform live-cell imaging due to space constraints.

Here, we propose a mirror-enhanced scanning LFM to achieve long-term high-speed 3D imaging at ~400 nm isotropic resolution with a single objective (40×/0.8NA). By simply placing a tilted mirror below the sample, we can achieve multiple-view LFM within a single image benefiting from the extended DOF. In addition to the improvement in the signal-to-noise ratio (SNR) by collecting more fluorescence reflected from the mirror, we achieved over threefold axial resolution improvement while maintaining the capability of high-speed 3D imaging with a compact system. A multiview phase-space deconvolution method was proposed to obtain high-resolution artifact-free 3D reconstruction directly from multiplexed images. To establish unique capabilities, we demonstrated various experiments in different types of photosensitive cells under extremely low light conditions. Moreover, we showed that the superior axial resolution facilitated much more robust 3D blood cell tracking in zebrafish larvae at the millisecond scale.

## Results

### MiSLFM substantially improves axial resolution

**Fig. 1:**
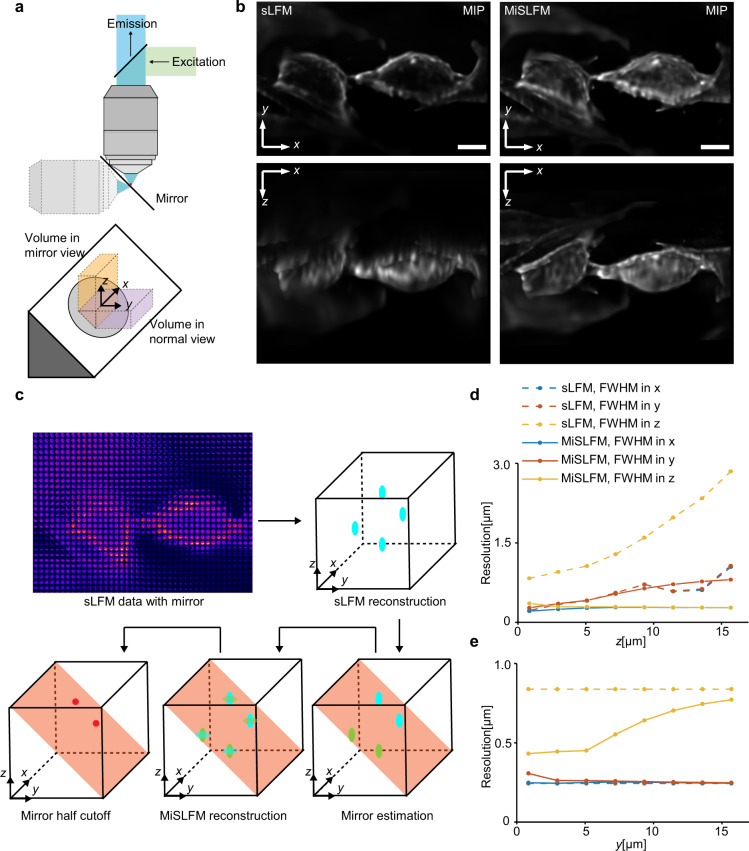
Principles and evaluations of MiSLFM. **a** Objective and mirror configurations of MiSLFM. The reference coordinate system on the mirror and a schematic of two light-field volumes are shown in light yellow (volume in mirror view) and lavender (volume in normal view), and the overlapping volume indicates the isotropic resolution volume. **b** Maximum intensity projections (MIPs) of reconstructed cell membrane-stained L929 cells for sLFM and MiSLFM, demonstrating an obvious axial resolution improvement for MiSLFM. Scale bars, 10 μm. **c** MiSLFM reconstruction pipeline. An initial 3D volume was reconstructed directly from the sLFM data including the mirror image, and a mirror estimation was applied to the volume. Then, the sLFM data were reconstructed again with the constraint of mirror symmetry, and only half of the volume was reserved. Coordinate systems in (**c**) are in concordance with the coordinate systems in (**a**). **d**, **e** Lateral and axial PSF FWHM for sLFM and MiSLFM estimated by simulations with a 40×/0.8NA objective along the z-axis and y-axis in the coordinate system of (**a**). MiSLFM improved the axial resolution across the entire overlapping volume and achieved a near-isotropic resolution of 0.4 μm near the mirror. The axial resolution gradually dropped when the sample moved away from the mirror along the y-axis

LFM suffers from the missing-cone problem^23,24^ with a severely degraded axial resolution, especially for low-NA objectives. As we can see in the results of fixed L929 cells imaged by sLFM^21,22^ shown in Fig. 1b and Fig. S1a), the maximum intensity projection (MIP) of the x-z plane shows significant blur along the z-axis, which makes it hard to distinguish a complete cell outline.

To improve the axial resolution and optical sectioning, we proposed MiSLFM, which simply adds a mirror to the traditional sLFM^21,22^ setup to provide a mirror image along the z-axis. Then, we added the symmetry constraint of the image and the mirror image in the phase-space deconvolution algorithm^32^. By culturing cells and tissue on the surface of a mirror with a protective 25-nm SiO_2_ coating for biocompatibility, we could multiplex the image and mirror image of the cells or tissue in a single light-field image, corresponding to the superposition of two orthogonal views (Fig. 1a, c). For 3D reconstruction, we made an initial 3D reconstruction directly from the sLFM data, leading to a 3D volume with symmetric structures. Then, we estimated the mirror position from the initial volume and updated the reconstruction results by adding the symmetry constraint of the image and the mirror image (see Methods). Compared to traditional sLFM, MiSLFM achieved a similar lateral resolution but with substantially improved axial resolution. The MiSLFM results showed a complete cell outline and clear membrane border in the x-z view. To quantitatively analyze the axial resolution improvement, we compared our MiSLFM with sLFM by imaging various subdiffraction-limited fluorescence beads with a 40×/0.8NA objective. We found that both MiSLFM and sLFM could achieve an ~0.4-μm lateral resolution, while MiSLFM maintained the same resolution in the axial domain. In contrast, sLFM could only achieve an axial resolution of ~1.5 μm (Fig. S2). For better visualization of the resolution improvement quantitatively, we simulated the acquisition and reconstruction of spheres at different 3D positions by sLFM and MiSLFM. The axial resolution of sLFM dropped rapidly with increasing distance to the mirror while that of MiSLFM remained similar (Fig. 1d). In addition, the axial resolution of MiSLFM gradually worsened along the y-axis but it was still much better than that of sLFM throughout (Fig. 1e). Then, we simulated the MiSLFM results of randomly distributed spheres with the mirror tilted at different angles (from 0° to 45°). We found that the axial resolution increased with increasing angle and finally achieved the same resolution as the lateral resolution at 45° (Fig. S3).

**Fig. 2:**
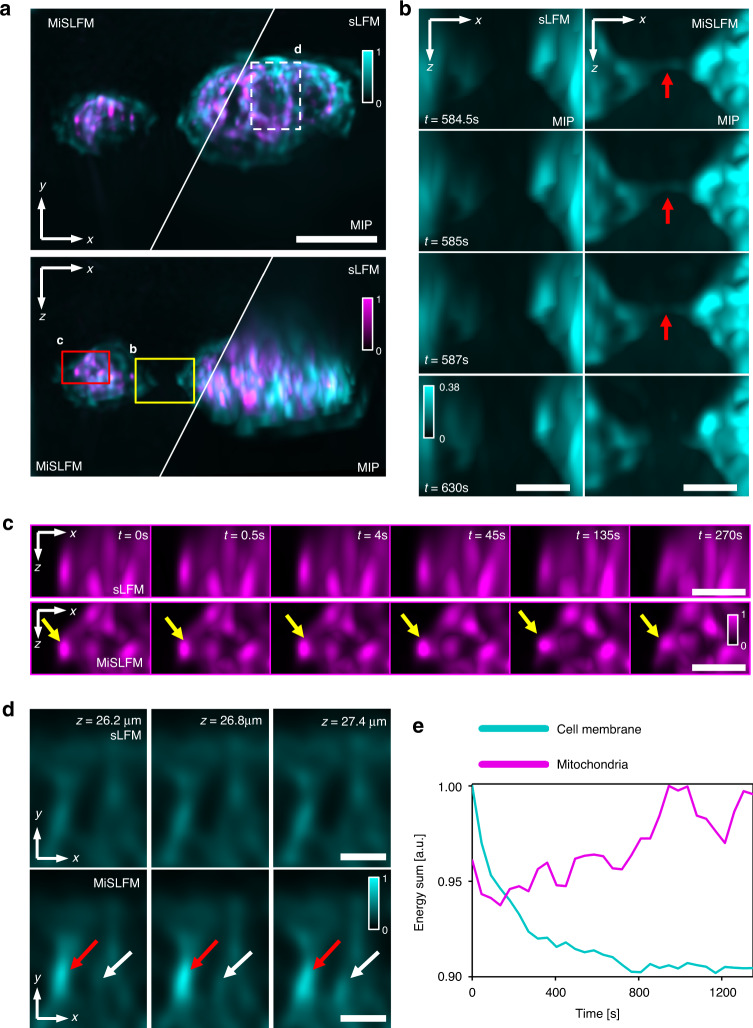
Two-color volumetric imaging of the membrane and mitochondria of NRK cells. **a** MIPs of the cell membrane (cyan, 488 nm) and mitochondria (magenta, 561 nm), at a 2-Hz volume rate over 90 µm × 70 µm × 70 µm reconstructed by sLFM and MiSLFM, respectively, under the laser power of 3.95 mW mm^−2^ (488 nm) and 1.99 mW mm^−2^ (561 nm), demonstrating the obvious axial resolution improvement for MiSLFM under a 40×/0.8NA objective. **b** Magnified areas marked by yellow boxes in (**a**) of the cell membrane MIP time series by sLFM (left) and MiSLFM (right). Red arrows mark the cell membrane contact of two cells, which is hard to visualize in traditional sLFM and LFM. **c** Magnified areas marked by red boxes in (**a**) of the mitochondrial MIP time series by sLFM (top) and MiSLFM (bottom). Yellow arrows mark the mitochondria. **d** Magnified slices marked by white dashed boxes in (**a**) at *z* = 26.2, 26.8, and 27.4 µm by sLFM (top) and MiSLFM (bottom). Red and white arrows mark locations with obvious intensity changes to show the improved capability of optical sectioning. **e** Normalized temporal traces of the whole two-color volumes in (**a**). No photobleaching in mitochondria and only 10% photobleaching in the cell membrane were observed over 20 min. Scale bars in **a** is 20 µm, in **b**–**d** are 5 µm

We further compared our MiSLFM with sLFM on NRK live cells by two-color imaging at 2 Hz in 3D (Fig. 2). The membrane is shown in cyan and the mitochondria are shown in magenta. We found that MiSLFM showed the same lateral resolution as sLFM in the MIP of the x-y plane, while MiSLFM showed significant axial resolution improvement in the MIP of the x-z plane (Fig. 2a). We selected two subregions of the x-z MIP and displayed their time series to demonstrate the axial resolution improvement. From the reconstruction results of MiSLFM (Fig. 2b), we observed the cell membrane contact between two NRK cells, which was marked by a red arrow. However, we could not observe any membrane contact between cells in the sLFM results because of the low axial resolution. In another channel, mitochondria also showed better axial resolution along with the entire time series (Fig. 2c), which is marked by a yellow arrow for the entire series. To analyze the optical sectioning ability of MiSLFM, we selected a subregion marked by a white dashed box in the MIP of the x-y plane (Fig. 2a). We found the x-y slice of the subregion showed little change from *z* = 26.2 μm to *z* = 27.4 μm in the sLFM results, while the x-y slice showed obvious intensity changes at different locations marked by red and white arrows in the MiSLFM results.

### MiSLFM facilitates high-speed 3D imaging with low phototoxicity

LFM is widely used in high-speed volumetric imaging due to its low photobleaching and phototoxicity^19,20^, compared to scanning methods, such as confocal microscopy, and spinning disk confocal microscopy. High-speed, light-sheet microscopy has low phototoxicity by illuminating only the in-focus information, but it is difficult to apply in various samples due to tissue opacity and space constraints. In contrast, LFM can achieve similar low phototoxicity by imaging the entire volumetric information within the extended DOF using a single objective and compact system. By introducing phase-space scanning to LFM, sLFM further pushes the 3D resolution to the diffraction limit of the whole objective NA^21^. Since sLFM collected the emitted fluorescent photons in the same way as LFM with high photon efficiency, sLFM shows very low photobleaching and phototoxicity. However, some emitted photons could not be collected in sLFM, such as the photons propagating opposite to the acquisition direction. By simply adding a mirror, MiSLFM could double the collected photons in theory, compared to sLFM. In addition, the sample is illuminated by a double intensity laser because of the mirror reflection. Therefore, MiSLFM could collect twice the number of photons with only half of the laser intensity. To demonstrate the good performance of MiSLFM in low photobleaching and phototoxicity, we imaged NRK live cells with fluorescence labeling on both membrane and mitochondria, which are easily bleached (Fig. 2). The input illumination density was set to be 3.95 mW mm^−2^ (488 nm) and 1.99 mW mm^−2^ 56 nm) for high-speed 3D imaging. We summed the fluorescence intensity in the current field of view (Fig. 2a) and plotted the fluorescence intensity curve over the recording time. We found that the mitochondrial channel showed no intensity downward trend and that the cell membrane channel showed only 10% photobleaching over 20 min.

**Fig. 3:**
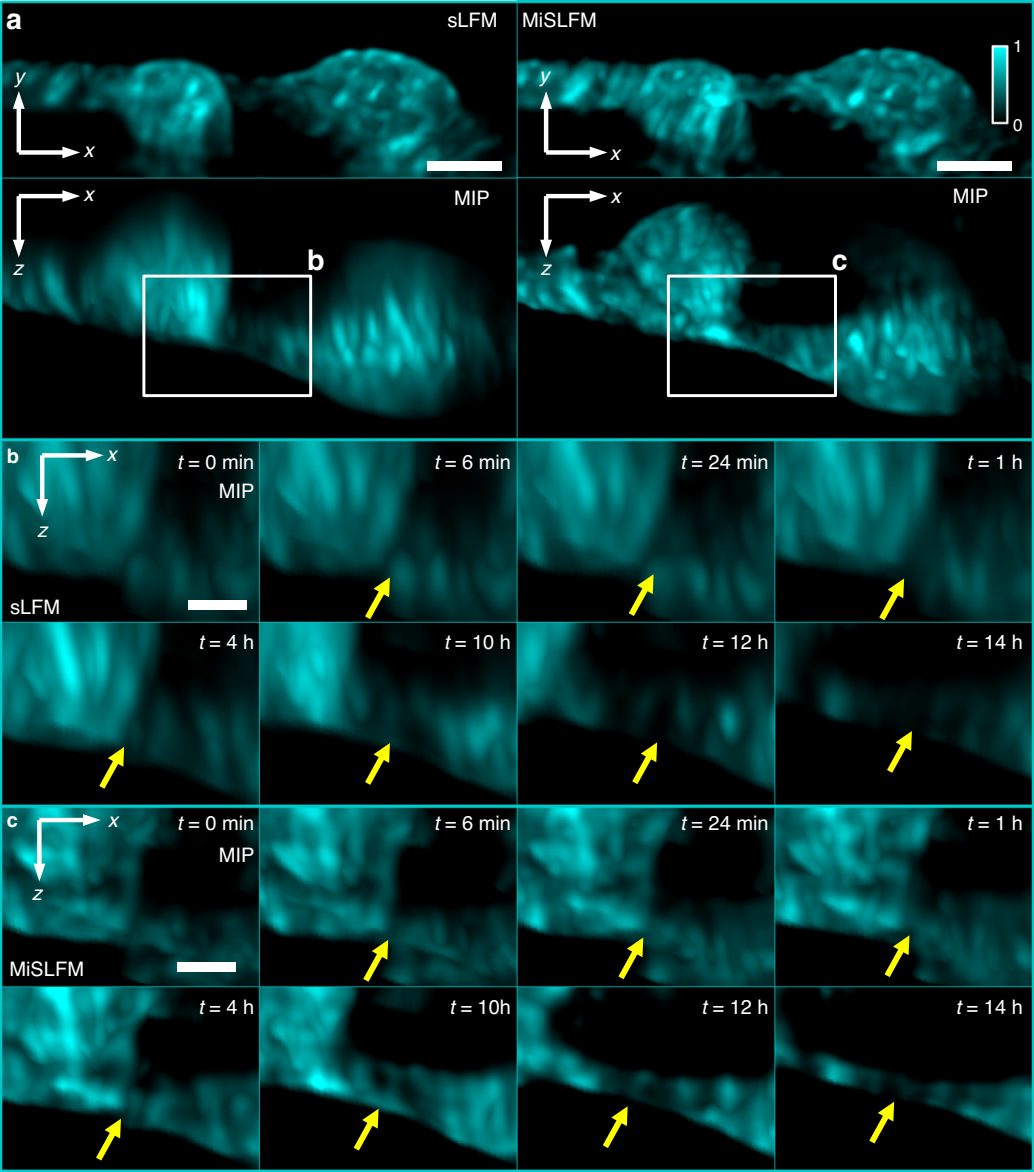
Long-term volumetric imaging of B16-GFP cells under extremely low light. **a** MIPs of the reconstructed membrane (cyan, 488 nm), at 6-min intervals (illumination time of 2.25 s) and up to 14 h of recording for sLFM and MiSLFM under 0.22 mW mm^−2^ illumination, demonstrating low photobleaching and phototoxicity for MiSLFM. **b**, **c** Magnified areas marked by white boxes in (**a**), by sLFM (**b**), and MiSLFM (**c**). Yellow arrows mark the cell membrane contact of two cells, which shows the superior axial resolution of MiSLFM and indicates that two cells maintain cell viability after 14 h of imaging. Scale bars in (**a**) is 10 µm, in (**b**) and (**c**) are 5 µm

We further demonstrated the superior performance of MiSLFM in long-term B16-GFP live-cell imaging (Fig. 3). We recorded B16 cells for up to 14 h with a time interval of 6 min by MiSLFM and up to 11 h with a time interval of 6 min by confocal microscopy for comparison. The input illumination density of MiSLFM for B16 cells was 0.22 mW mm^−2^ (488 nm) and the illumination time was 2.25 s at 6-min recording intervals. For confocal microscopy, we kept the same recording interval and image scale under a 0.44 mW mm^−2^ illumination density, but the illumination time was much longer since confocal microscopy is a point scanning method with low 3D imaging speed. We found that MiSLFM achieved better axial resolution and almost the same lateral resolution compared to sLFM (Fig. 3a). The axial resolution improvement also maintained good consistency across different time frames (Fig. 3b, c). Generally, the better the cell viability is, the flatter the cell. The gradual rounding of the cells means that the cell viability worsens. When the cells become rounded, the contact between the cells disappears. We found that the two cells shown in the MiSLFM results remained in contact with each other for at least 14 h (Fig. 3c). However, the two cells shown in the confocal results could only be imaged within 3 h (Fig. S4). MiSLFM showed much better performance on low phototoxicity than confocal microscopy. In addition, by plotting the fluorescence intensity curve over the recording time under their respective fields of view, we found that there was almost no photobleaching in the MiSLFM results but more than 90% intensity attenuation according to the confocal microscopy results over 10 h.

### MiSLFM achieves performance robustness on fast-moving samples

**Fig. 4:**
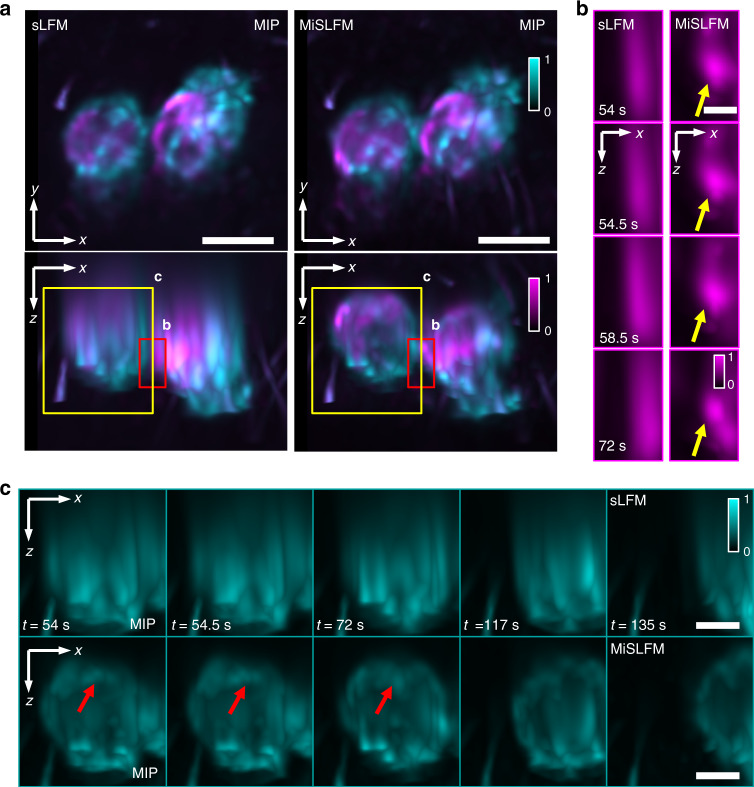
Two-color volumetric imaging of contractile vacuoles and the cell membrane of Dictyostelium discoideum. **a** MIPs of the reconstructed contractile vacuole (magenta, 488 nm) and cell membrane (cyan, 561 nm), at a 2-Hz volume rate over 50 µm × 50 µm × 40 µm for sLFM and MiSLFM under 1.71 mW mm^−2^ (488 nm) and 1.55 mW mm^−2^ (561 nm) illumination, demonstrating axial resolution improvement for MiSLFM under a 60×/1.1 NA objective. **b** Magnified areas marked by red boxes in (**a**) by sLFM (left) and MiSLFM (right). Yellow arrows mark the vesicles, which show precise axial localizations in MiSLFM. **c** Magnified areas marked by yellow boxes in (**a**) by sLFM (top) and MiSLFM (bottom). Red arrows mark a point in the upper cell membrane movement, which could not be observed in sLFM and could be clearly visualized in MiSLFM. Scale bars in (**a**) is 10 µm, in (**b**) is 2 µm, in (**c**) is 5 µm

To confirm the robustness of our method to fast-moving photosensitive samples, we conducted an experiment on Dictyostelium discoideum (GFP labeled contractile vacuoles and mRFP labeled cell membranes with a 60×/1.1 NA water-immersion objective and a 33-degree tilted mirror at 2 Hz volume rate. Dictyostelium discoideum is very sensitive to light and usually moves very fast, which is difficult for traditional methods to image. The cell membrane is shown in cyan, and the contractile vacuole is shown in magenta. In this experiment, Dictyostelium discoideum crawled freely on the surface of the mirror. The input illumination densities for MiSLFM were 1.71 mW mm^−2^ (488 nm) and 1.55 mW mm^−2^ (561 nm). We found that MiSFLM showed the same lateral resolution as sLFM on the MIP of the x-y plane, with significant axial resolution improvement and artifact reduction on the MIP of the x-z plane (Fig. 4a). The reconstructed volume size here was ~50 μm × 50 μm × 40 μm. Although the 33-degree mirror could not achieve isotropic resolution, which made the axial resolution slightly worse than the lateral resolution, it still achieved considerable improvement (Fig. S3). We selected two subregions on the MIP of the x-z plane and displayed their time series to demonstrate the axial resolution improvement. We could distinguish the movement of the contractile vacuole in the axial direction benefiting from the axial resolution improvement by MiSLFM, which is marked by yellow arrows (Fig. 4b). We recorded the rapid movement of Dictyostelium discoideum on the surface of the mirror for ~3 min. The Dictyostelium discoideum (marked by yellow boxes) underwent rapid migration within 1 min (Fig. 4c). During this process, the superior axial resolution of MiSLFM enabled recording the movement of the upper cell membrane of the Dictyostelium discoideum which is marked by red arrows (Fig. 4c), while traditional sLFM could only resolve cell membrane movement close to the mirror. In summary, MiSLFM improved the axial resolution while maintaining high-speed imaging for rapidly changing scenes.

### MiSLFM facilitates robust tracking of blood cells in zebrafish larvae

**Fig. 5:**
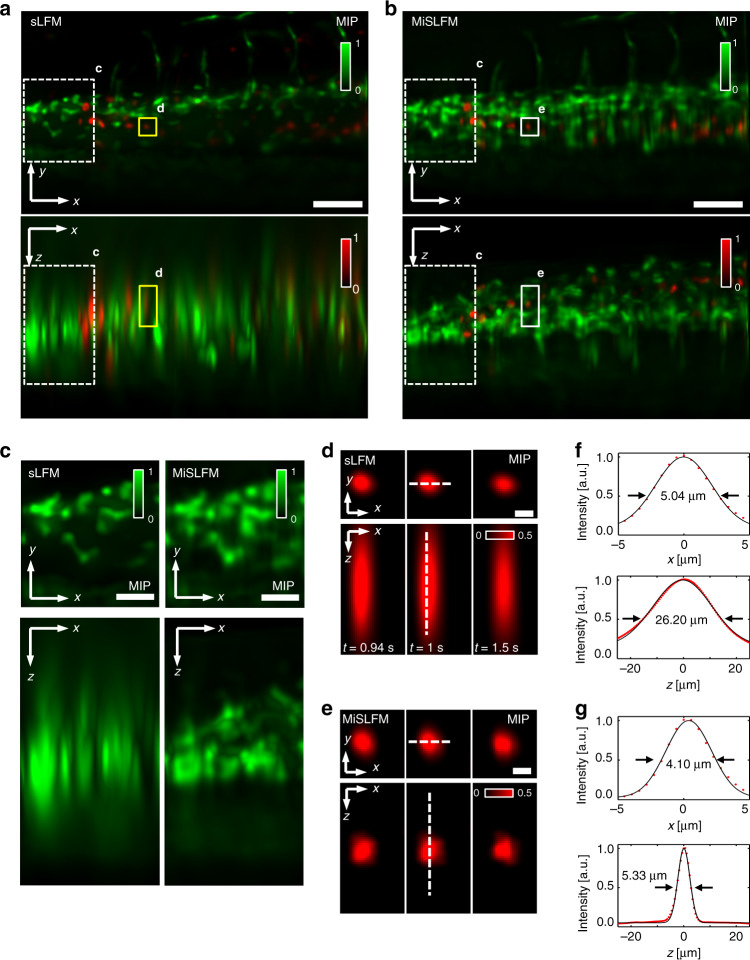
High-speed volumetric imaging of zebrafish blood flow under low-illumination power. **a**, **b** MIPs of reconstructed blood vessels (green, 488 nm) and blood cells (red, 561 nm), for sLFM and MiSLFM under 0.54 mW mm^−2^ (488 nm) and 0.71 mW mm^−2^ (561 nm) illumination, demonstrating an obvious axial resolution improvement for MiSLFM under a 20×/0.5 NA objective. The blood vessels were only imaged once and the blood cells were imaged at an 18-Hz volume rate. **c** Magnified areas marked by white dashed boxes in (**a**, **b**) by sLFM (left) and MiSLFM (right). **d**, **e** Magnified areas marked by yellow and white boxes in (**a**, **b**) by sLFM (top) and MiSLFM (bottom), which shows that the axial resolution improvement indicates good consistency across different time frames for MiSLFM. **f**, **g** Intensity profile along the white dashed line in (**d**, **e**), by sLFM (**f**), and MiSLFM (**g**), demonstrating an at least fivefold improvement in axial resolution. Scale bars in (**a**) and (**b**) are 50 µm, in (**c**) is 20 µm, in (**d**) and (**e**) is 5 µm

Finally, we demonstrated that the superior axial resolution of MiSLFM could facilitate robust blood cell imaging and cell tracking in vivo in zebrafish larvae at high speed with low-illumination density. The blood vessels are shown in green and the blood cells are shown in red. We embedded 3 dpf-anesthetized zebrafish in 0.4% low-melting-temperature agarose and observed them with a 20×/0.5 NA water-immersion objective. The input illumination densities here were 0.54 mW mm^−2^ (488 nm) and 0.71 mW mm^−2^ (561 nm). We first imaged the blood vessels once and then imaged blood cell movement at an 18 Hz volume rate. The acquisition frame rate of the camera is 18 Hz with 3×3 scanning for sLFM. By using the sliding window method (window size is 9) combined with the time-weight algorithm, we can compensate for the loss of temporal resolution up to the camera frame rate. We reconstructed an area of ~90 μm × 50 μm × 70 μm that contained blood vessels and blood cells in the zebrafish tail (Fig. 5a, b). By superimposing these two channels afterward, we showed a two-color zebrafish image with both blood vessel walls and running blood cells. We found that MiSLFM continued to show a substantial axial resolution improvement over sLFM with similar lateral resolution (Fig. 5a–c). MiSLFM made the axial distribution of blood vessel membranes clearly identifiable while traditional sLFM could not distinguish the distribution of blood vessels in the axial direction (Fig. 5c). However, the speed of sLFM could keep up with the blood cell flow of anesthetized zebrafish (Fig. 5d, e). Our proposed method achieved isotropic resolution for blood cells (Fig. 5e, g), while traditional sLFM showed an elongated distribution in the axial domain (Fig. 5d, f). Furthermore, we identified and tracked the blood cells with Imaris software by using its built-in automatic tracking algorithm (Fig. S5). Compared to sLFM, we detected more blood cells imaged by MiSLFM. Specifically, eight cells and two cells were detected in the MiSLFM and sLFM results respectively, demonstrating that our proposed method could significantly promote robust high-speed blood cell imaging and cell tracking.

## Discussion

In summary, we have demonstrated a highly efficient and compact imaging method to obtain long-term high-speed 3D subcellular imaging at the isotropic resolution, which can be a compact add-on for any normal upright wide-field fluorescence microscope. By multiplexing multiple views with a simple mirror beneath the sample, we fully exploited the extended DOF of LFM to address the missing-cone problem with a single objective without sacrificing the 3D imaging efficiency. With these unique capabilities, we achieved long-term millisecond-scale 3D fluorescence imaging at ~400 nm isotropic resolution with extremely low light illumination. In our system, we use a multimode fiber laser to avoid the interference of the incident and reflected light for illumination. And due to the short coherence length of fluorescent molecules with a broad bandwidth, even the fluorophores close to the mirror do not show apparent interference patterns. Compared to the traditional LFM, our method MiSLFM sacrifices the FoV to obtain more measurements for the mirror image and improve the axial resolution. Besides this trade-off, the resolution will decrease when the sample is located close to the edge of the FoV. LFM with multiple objective lenses in open-top format^31^ can observe large-scale samples conveniently, while the resolution is not enough for subcellular structures. A tilted imaging framework may be developed in the future to simplify the procedures for sample preparation in MiSLFM.

Traditional imaging schemes capture 3D information in real space directly using complicated optical systems for optical sectioning, aberration correction, or 3D mapping. In this case, too much pressure is imposed on the optical system with inevitable tradeoffs in the resolution, phototoxicity, and speed, especially when the applications do not require real-time high-quality feedback. In contrast, we pack the photons in phase space with simple optics to collect multiplexed multiview data at high speed in an efficient, robust, and flexible way. Then, we leave the reconstruction of native high-resolution 3D information to computing with the repeated usage of high-resolution phase-space measurements at different depths. The higher efficiency in both sampling and reconstruction, as well as the reflected fluorescence from the mirror, significantly increases the SNR and the resolution. Consequently, we believe such improvements in both SNR and spatiotemporal resolution will bring advanced imaging capability via low-cost compact systems to the broad biology community, facilitating robust and quantitative analysis in various applications such as organelle interactions, cellular vesicular transports, and intercellular communications.

## Materials and methods

### MiSLFM optical setup

The optical setup consists of a commercial Olympus upright microscope (BX43), a laser arm for fluorescence illumination, and an optical collection arm. The objective lens used in the experiment included a 20×/0.5 NA (Olympus, Cat. #UMPLFLN20XW) water-immersion objective, a 40×/0.8NA (Olympus, Cat. #LUMPLFLN40XW) water-immersion objective, and a 60×/1.1 NA (Olympus, Cat. #UMPLFLN20XW) water-immersion objective. The light source used for fluorescence excitation was a switchable continuous multimode fiber laser (*λ* = 488 nm and *λ* = 561 nm, Oxxius, Cat. #L4Cc; *λ* = 640 nm, 89North, Cat. #LDI-7). In the following optical path, the system included a relay lens pair with an optical magnification of 1.15×; thus, the final equivalent magnification was 23×, 46×, and 69× for the objectives. A two-dimensional piezo (Coremorrow, Cat. #P33.T2S) was inserted at the pupil plane of the 4 F system, and the microlens array (pitch size of 97.5 µm with a focal length of 1.95 mm) was placed at the image plane of the 4 F system. After that, with a second 1:1 optical relay lens pair, an sCMOS camera (PCO Panda4.2, 2048 × 2048 pixels) was placed at the conjugated focal plane of the MLA (Fig. S6). The excitation light path was coupled to an upright microscope with a four-color filter set (405/488/561/640 nm, Chroma, 89901v2).

### Data processing

The reconstruction pipeline includes PSF generation, phase-space deconvolution, and mirror estimation (Fig. S7). The PSF generation and phase-space deconvolution process was based on the Lu, Z. et al. algorithm^32^, and we combined these methods with mirror estimation and mirror symmetry constraints to achieve multiview reconstruction. We first estimated the aberration of the system by capturing the fluorescent beads to generate the system PSF for different objectives. Subsequently, we conducted an initial 3D reconstruction directly from sLFM data and used the reconstructed volume to estimate the position of the fixed-angle mirror in the reconstructed volume. When the estimated value of *θ* is within 0.5° of the accurate value, it will not affect the accuracy of the 3D reconstruction results (Fig. S8). Then, we updated the reconstruction results by adding the symmetry constraint of the image and the mirror image. The detailed pipeline is divided into six parts: PSF generation, sLFM deconvolution, mirror modeling, volume warping, mirror estimation, and mirror-enhanced deconvolution (details shown in Supplementary Note 1). The whole process was performed on a normal computer (Intel i9-9980XE CPU, RTX Quadro 8000 GPU, and 128 GB memory), with a reconstruction script in MATLAB r2019a. Reconstruction times depended on the PSF size, iteration times, and selected ROI size of the data, typically, it needs ~9 min for an ~500 × 500 × 500 voxel volume with ten iterations.

### Custom design of sample chamber and mirror

In MiSLFM, a custom-designed sample chamber and a piece of a commercial mirror were employed in place of the microscope slide or petri dish. The 3D design and physical overview of the custom-designed sample chamber are shown in Fig. S9. The length and width of the chamber were 75 mm × 30 mm so that the chamber could match well with the sample holder on the stage of a commercial upright microscope, such as the Olympus BX43. The angle of the slope where the mirror is placed depends on the angle of the objective. For the water-dipping objectives UMPLFLN20XW (Olympus) and LUMPLFLN40XW (Olympus), the angle was 45°. However, for objective LUMFLN60XW (Olympus), the angle was only 33°. We give priority to ensuring that the NA of the objective will not be affected (Fig. S10). For the objective with large NA and a short working distance, our strategy following the above principle will slightly reduce the axial resolution, but the axial resolution is still greatly improved as shown in Fig. 4. The 3D design drawings of the sample chamber are available from the corresponding authors upon reasonable request. The commercial mirror used was a first-surface mirror (#87-367, Edmund Optics), with a protective 25-nm SiO_2_ coating that is biocompatible for cell culture and tissue attachment after pretreatment with fibronectin or polylysine. The thickness of protective SiO_2_ coating is around 25 nm, which has been taken into consideration for 3D reconstructions.

### Sample preparation

#### L929 fluorescent staining and imaging

A total of 3 × 10^4^ L929 cells used in this study (Fig. 1 and Fig. S1) were seeded on human fibronectin (200X diluted with 1X PBS) pretreated SiO_2_ coated mirrors and returned to a CO_2_ incubator for 24 h. Then, the cells were fixed with 4% paraformaldehyde, washed with PBS three times, stained with WGA-647 (Thermal Fisher, 500X diluted with 0.05% Tween 20 in 1X PBS) for 2 h at room temperature, and protected from light. Afterward, the cells were thoroughly washed with 1X PBS three times and then transferred to a custom-designed sample chamber filled with 1X PBS, and imaged with LUMPLFLN40XW (Olympus) at room temperature.

#### NRK fluorescent live-cell recording

A total of 3 × 10^4^ NRK cells (TSPAN4-GFP/Mito-DsRed) used in this study (Fig. 2) were seeded on human fibronectin (200X diluted with 1X PBS) pretreated SiO_2_ coated mirror and returned to a CO_2_ incubator for 24 h. On the next day, the cell-seeded mirror was directly transferred to the custom-designed sample chamber and submerged in DMEM supplemented with 10% FBS and 1% Pen/Strep antibiotics. Then, live cells labeled with GFP and DsRed were recorded at a 2-Hz volume rate with LUMPLFLN40XW (Olympus) at 37 °C.

#### B16-GFP long-term fluorescent live-cell recording

GFP-expressing B16F10 cells (5 × 10^4^) were seeded on the SiO_2_ coated mirror and returned to the CO_2_ incubator for 24 h. On the next day, the cell-seeded mirror was directly transferred to the custom-designed sample chamber and submerged in DMEM supplemented with 10% FBS and 1% Pen/Strep antibiotics. For long-term fluorescent live-cell recording, a heating pad was placed beneath the custom-designed sample chamber to keep the cell culture medium at 37 °C throughout the entire recording procedure, and a thin layer of mineral oil was applied to cover the surface of the DMEM to prevent culture medium evaporation. The live-cell GFP fluorescence of B16F10 cells was recorded with LUMPLFLN40XW (Olympus) at a 6-min interval for 24 h.

#### Live-cell imaging of Dictyostelium discoideum

The Dictyostelium discoideum (dajumin-GFP/myr-mRFP) used in this study (Fig. 4) was labeled with contractile vacuoles (GFP) and cell membranes (mRFP). The mirror was coated with 1:100 diluted polylysine (Sigma, P4707, 0.01%) in 1× PBS for an hour, and then washed with 1× PBS three times. Dictyostelium discoideum (2 × 10^4^) was seeded on the SiO_2_-coated mirror. After 1 min, the Dictyostelium discoideum seeded mirror was directly transferred to the custom-designed sample chamber and submerged with water. Then, live-cell GFP and RFP fluorescence were recorded with a LUMFLN60XW (Olympus) at a 2-Hz volume rate for several minutes.

#### In vivo imaging of blood flow in zebrafish larvae

Zebrafish from the transgenic line Tg (gata1:DsRed) were crossed with zebrafish from the transgenic line Tg (flk: EGFP), and F1 larval zebrafish were used for blood cell imaging (Fig. 5 and Fig. S5). The embryos were raised in 165 mg l^−1^ 1-phenyl-2-thiourea (PTU) in embryo rearing medium (ERM) at 28.5 °C until 3 dpf larval development. Larval zebrafish were paralyzed by short immersion in 1 mg/ml α-bungarotoxin solution (Invitrogen). Afterward, the larvae were embedded in 0.4% low-melting-temperature agarose on the mirror surface. The larval zebrafish were recorded at room temperature using the UMPLFLN20XW (Olympus) at 18 Hz.

## Supplementary information

supplementary figures and tables of MiSLFM

Zebrafish Blood vessel walls and cells

NRK cells

Dictyostelium discoideum
