# Supplementary material for: Mirror-enhanced scanning light-field microscopy for long-term high-speed 3D imaging with isotropic resolution
Source: Light Sci Appl. 2021 Nov 4;10:227. doi: 10.1038/s41377-021-00665-9 (PMC8568963; doi:10.1038/s41377-021-00665-9)
Supplement: Supplementary file 1 — supplementary figures and tables of MiSLFM [file 41377_2021_665_MOESM1_ESM.pdf]

Supplementary Information for

Mirror-enhanced scanning light-field microscopy for long-term high-speed 3D imaging with isotropic resolution

Authors

Bo Xiong[1,2,3†], Tianyi Zhu[1,2,3†], Yuhan Xiang[4], Xiaopeng Li[4], Jinqiang Yu[4], Zheng Jiang[4], Yihan Niu[1,2,3], Dong Jiang[4], Xu Zhang[5], Lu Fang[2,6*], Jiamin Wu[1,2,3*], and Qionghai Dai[1,2,3*]

Affiliations

[1] Department of Automation, Tsinghua University, Beijing 100084, China.

[2] Institute for Brain and Cognitive Sciences, Tsinghua University, Beijing 100084, China.

[3] Beijing Laboratory of Brain and Cognitive Intelligence, Beijing Municipal Education Commission.

[4] State Key Laboratory of Membrane Biology, Tsinghua University-Peking University Joint Centre for Life Sciences, Beijing Frontier Research Center for Biological Structure, School of Life Sciences, Tsinghua University, Beijing 100084, China.

[5] Beijing Institute of Collaborative Innovation, Beijing 100094, China.

[6] Department of Electronic Engineering, Tsinghua University, Beijing 100084, China.

[†] These authors contribute equally to this work.

[*] Correspondence and requests for materials should be addressed to qhdai@mail.tsinghua.edu.cn (Qionghai Dai), wujiamin@tsinghua.edu.cn (Jiamin Wu) and fanglu@mail.tsinghua.edu.cn (Lu Fang)

Supplementary Information Table

**Supplementary Figure 1**	3D imaging of fluorescent stained L929 cell by sLFM and MiSLFM.
**Supplementary Figure 2**	Reconstructions of fluorescent beads by sLFM and MiSLFM and their resolution analysis.
**Supplementary Figure 3**	Comparison of MiSLFM reconstructions under different angle between the mirror and the x-y plane.
**Supplementary Figure 4**	Long-term, low illumination density volumetric imaging of B16-GFP cell by confocal microscopy.
**Supplementary Figure 5**	Zebrafish blood-cell tracking comparison between sLFM and MiSLFM.
**Supplementary Figure 6**	Schematic 3D drawings of the MiSLFM setup
**Supplementary Figure 7**	Reconstruction pipeline for MiSLFM.
**Supplementary Figure 8**	Comparison of MiSLFM reconstructions with error estimation
**Supplementary Figure 9**	Diagram and photogram of the sample chamber and mounting.
**Supplementary Figure 10**	Schematic diagram of angles for objective, sample chamber and effective NA
**Supplementary Table 1**	Optical objective and illumination source usages in experiments.
**Supplementary Note 1**	A Detailed protocol of reconstruction pipeline of MiSLFM.
**Supplementary Note 2**	Resolution artifacts analysis under MiSLFM reconstruction.
**Supplementary Video 1**	Comparison between sLFM and MiSLFM reconstruction of high-speed zebrafish blood flow volumetric imaging.
**Supplementary Video 2**	Comparison between sLFM and MiSLFM reconstruction of two-color volumetric imaging of NRK cells.
**Supplementary Video 3**	Comparison between sLFM and MiSLFM reconstruction of two-color volumetric imaging of Dictyostelium Discoideum.

Supplementary Figure 1

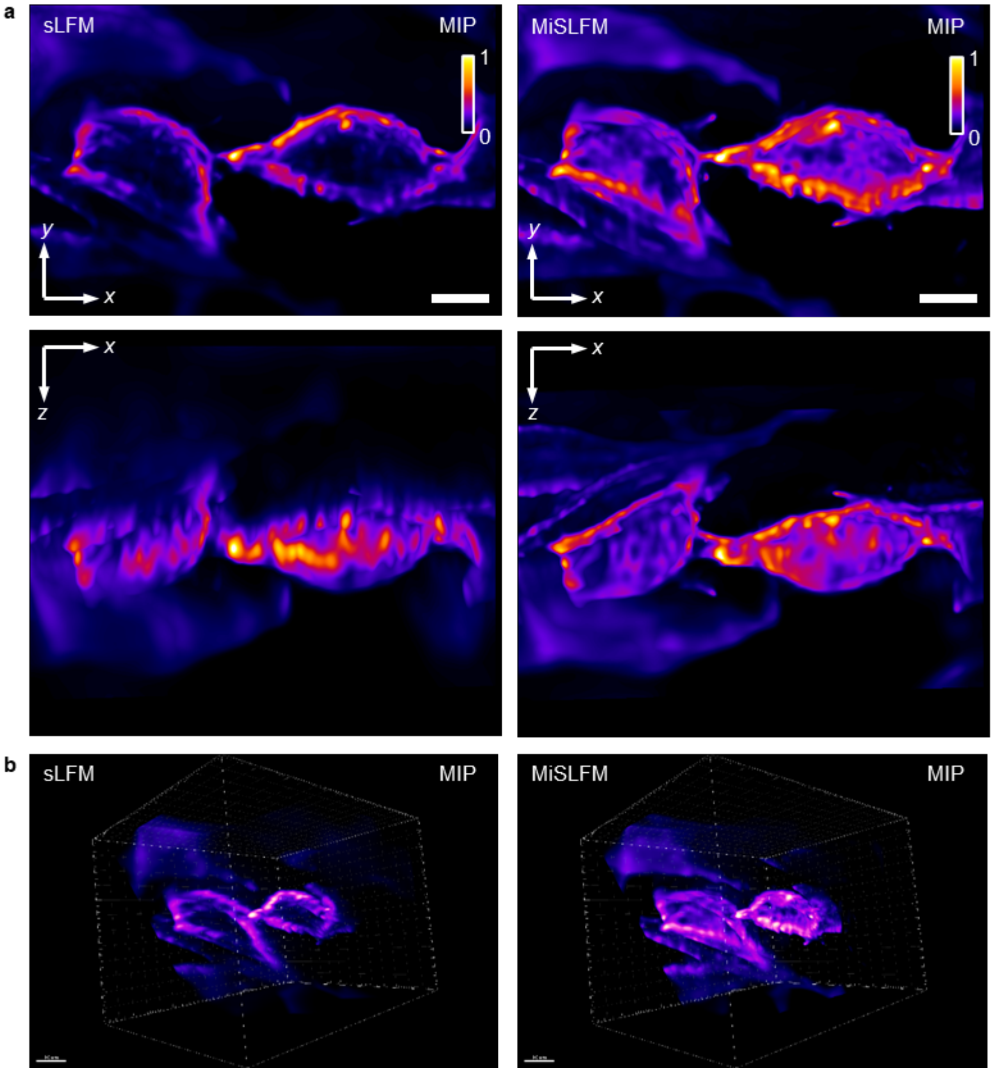

Volumetric imaging of fluorescent stained L929 cell by sLFM and MiSLFM.

(a) Maximum intensity projections of reconstructed cell membrane staining L929 cells for sLFM and MiSLFM, demonstrating an obvious axial resolution improvement for MiSLFM.
(b) Reconstructed cell membrane staining L929 cells by sLFM(left) and MiSLFM(right) rendered in 3D, demonstrating complete 3d outline of cells could be reconstructed by MiSLFM.
Scale bars, 10 μm.

Supplementary Figure 2

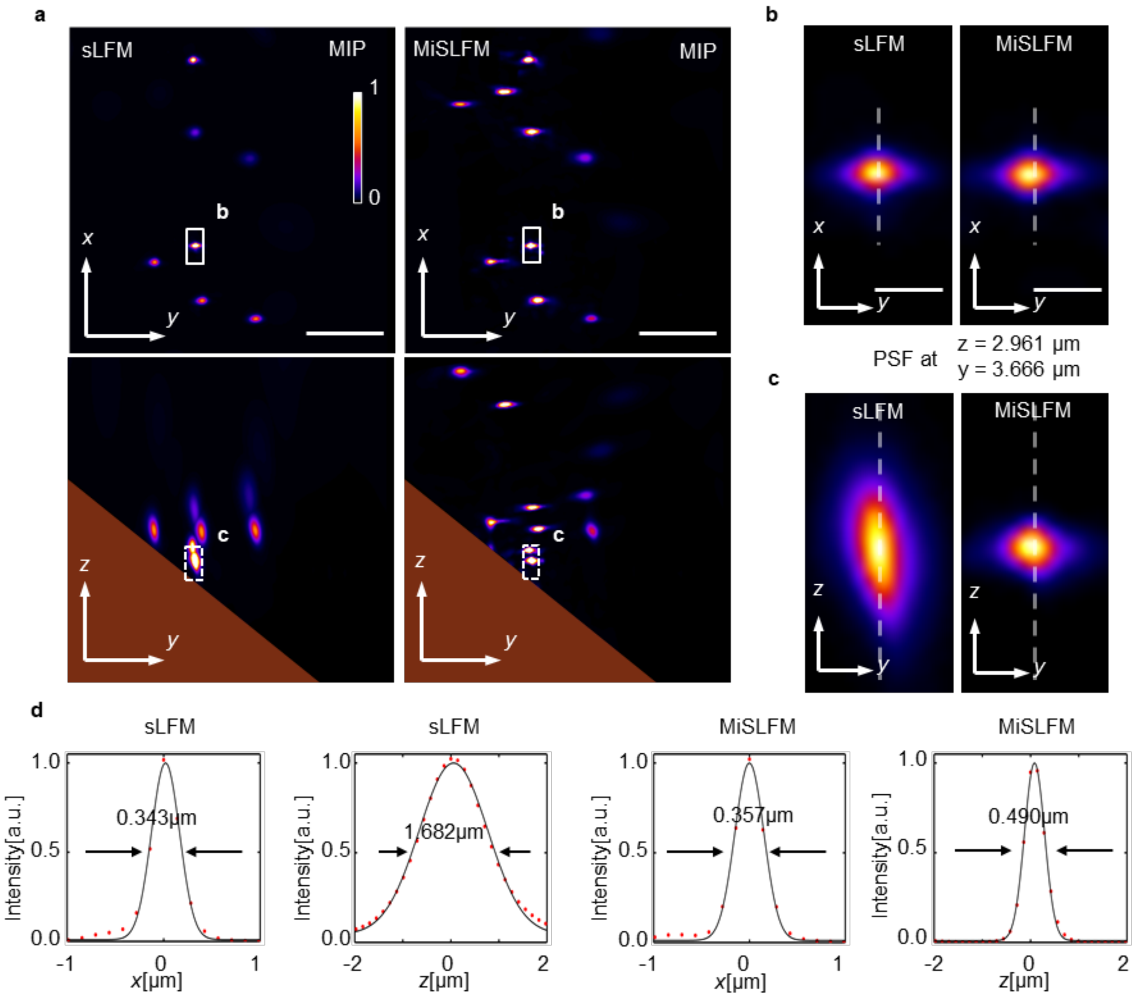

Reconstructed fluorescent beads and their respective resolution (FWHM) for sLFM and MiSLFM
(a) Maximum intensity projections of reconstructed fluorescent beads for sLFM and MiSLFM.
(b, c) zoomed MIP images from reconstructed volume of (a) marked by white boxes and white dashed boxes, the selected region size from (a) is 4.23×4.23×8.46 μm³.
(d) Intensity profile along the white dashed line in (b, c) by sLFM (left two subfigures) and MiSLFM (right two subfigures), demonstrating an approximately threefold improvement in axial resolution for MiSLFM.
Scale bars in (a) is 10 μm, in (b) and (c) are 1 μm.

Supplementary Figure 3

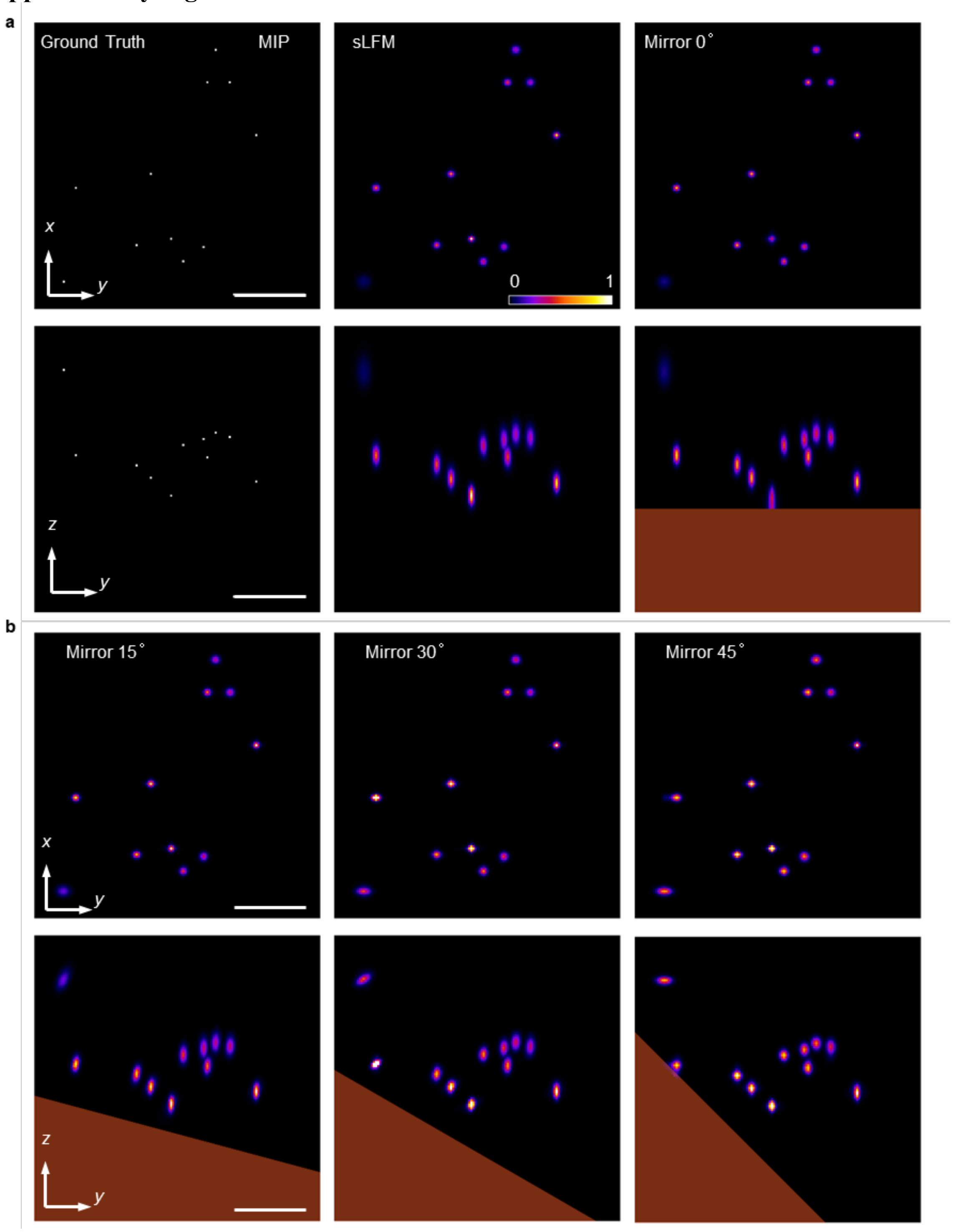

Comparison of MiSLFM reconstructions with mirror tilted at different angles.

(a) Reconstructions of a simulated 3D sample with randomly distributed beads with mirror tilted at different angles by sLFM and MiSLFM. First row from left to right is the ground truth of the simulated 3D sample with randomly distributed beads, reconstructions by sLFM, and reconstructions by MiSLFM with mirror tilted at 0-degree.

(b) Second row from the left to right is reconstructions by MiSLFM with mirror tilted at 15-degree, 30-degree, and 45-degree. As the angle increase, the axial resolution increase and become the same as lateral resolution with mirror tilted at 45-degree in the area close to the mirror.
Scale bars in (a) and (b) are 5 μm.

Supplementary Figure 4

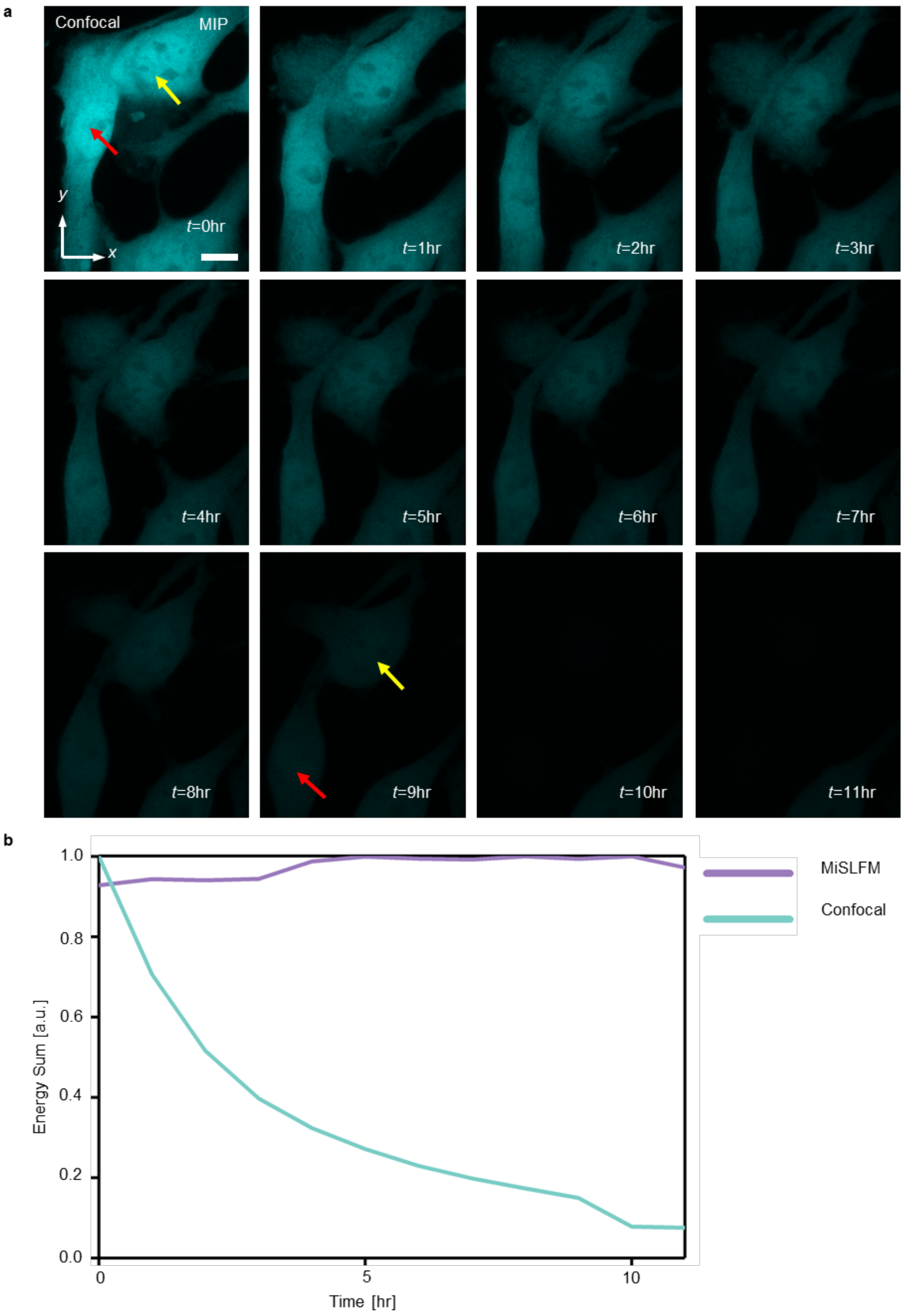

Long-term volumetric imaging of B16-GFP cell under low illumination density by confocal microscopy

(a) Maximum intensity projections of the B16 cell membrane (cyan 488nm), at 6 minutes recording interval (illumination time 300 s) and up to 11 hours recording for confocal microscopy under 0.44 $\mathrm{mW\ mm^{-2}}$ illumination. Red and yellow arrows mark two cells which contact each other at the beginning and then gradually get rounded darkened, indicating that two cells lose cell viability within 9 hours.
(b) Fluorescence intensity profile of confocal microscopy and MiSLFM, corresponding to their respective field of view, demonstrating confocal microscopy suffers from severe photobleaching (Ninety percent attenuation after 10 hours imaging).
Scale bars in (a) is 10 μm.

Supplementary Figure 5

a
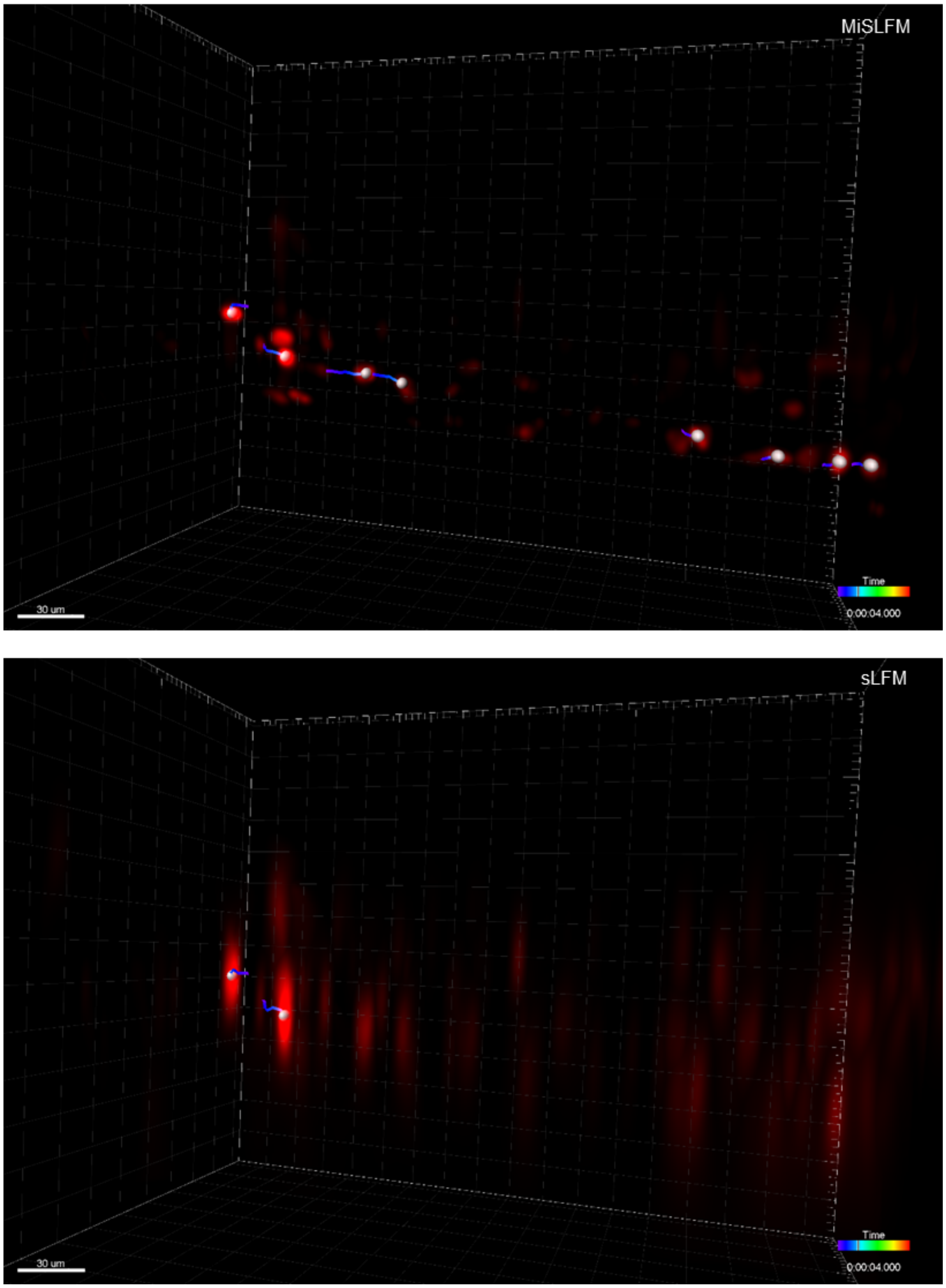

b

Blood-cell tracking through Imaris 9.0.1 software in low-power illumination dosage.

(a) Cell tracking results of reconstructed zebrafish blood cell by MiSLFM, which is the same experiment in Fig. 5. The overall tracing time length is 14.5 s. The total number of tracked cells is 8, which is automatically decided by the tracking algorithm in the software. Cell traces are color-coded by different time stamps and tracked cells are overlaid by white spheres.

(b) The same as (a) but by sLFM. The total number of tracked cells is 2.

Scale bars are 40 μm. Color bars show different time stamps.

Supplementary Figure 6

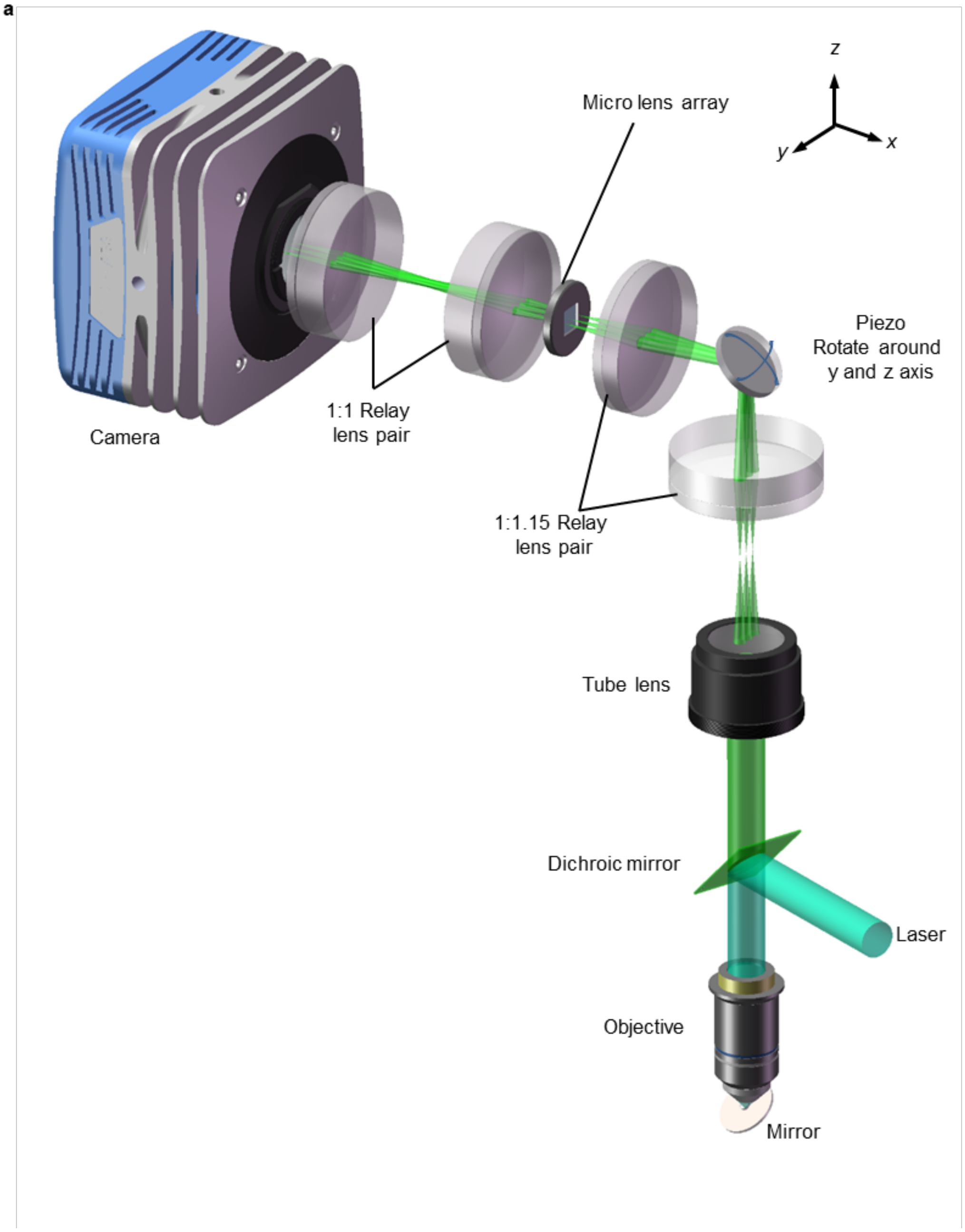

Mirror-enhanced scanning light-field microscopy (MiSLFM) setup.

(a) Schematic 3D drawings of the setup showing the main optomechanical components. 3D distributed samples seeded on the SiO_2 coated mirror will be collected by a standard upright microscope (i.e., an objective and a tube lens). The image of the standard microscope is conjugated to the microlens array (MLA) by 1:1.15 relay lens pair and a two-dimensional piezo is inserted into the Fourier plane of the first relay lens to generate image shift on the MLA along the y-axis and z-axis. Then, the image is coded by the MLA and captured by a camera with a 1:1 relay lens pair.

Supplementary Figure 7

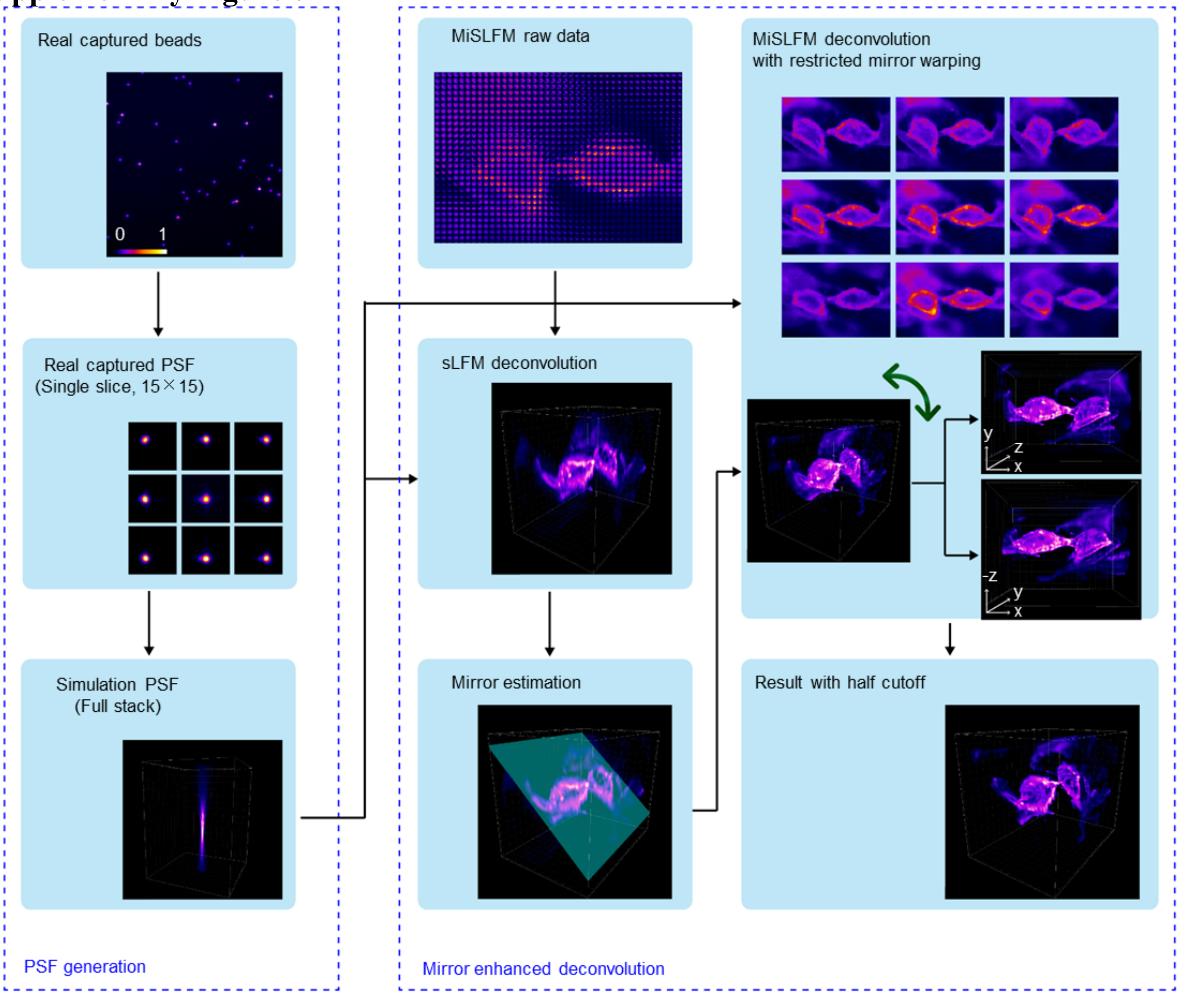

MiSLFM reconstruction pipeline.

The workflow for reconstruction the acquired scanning light-field(sLFM) data to isotropic resolution deconvolved 3D volumes. The reconstruction pipeline mainly includes two parts: PSF generation and Mirror enhanced deconvolution. First, a simulated aberrated PSF is generated based on the real capture beads. Then, the simulated PSF is used in the phase-space deconvolution of acquired scanning light-field data to get a rough 3D volume, and a mirror estimation is applied to the rough 3D volume. Finally, the sLFM data is reconstructed again with 3d volumes is constrained to mirror symmetry, and only half of the volume is reserved

The pipeline is based on MATLAB and details of the pipeline protocol are in Supplementary Note 1.

Supplementary Figure 8

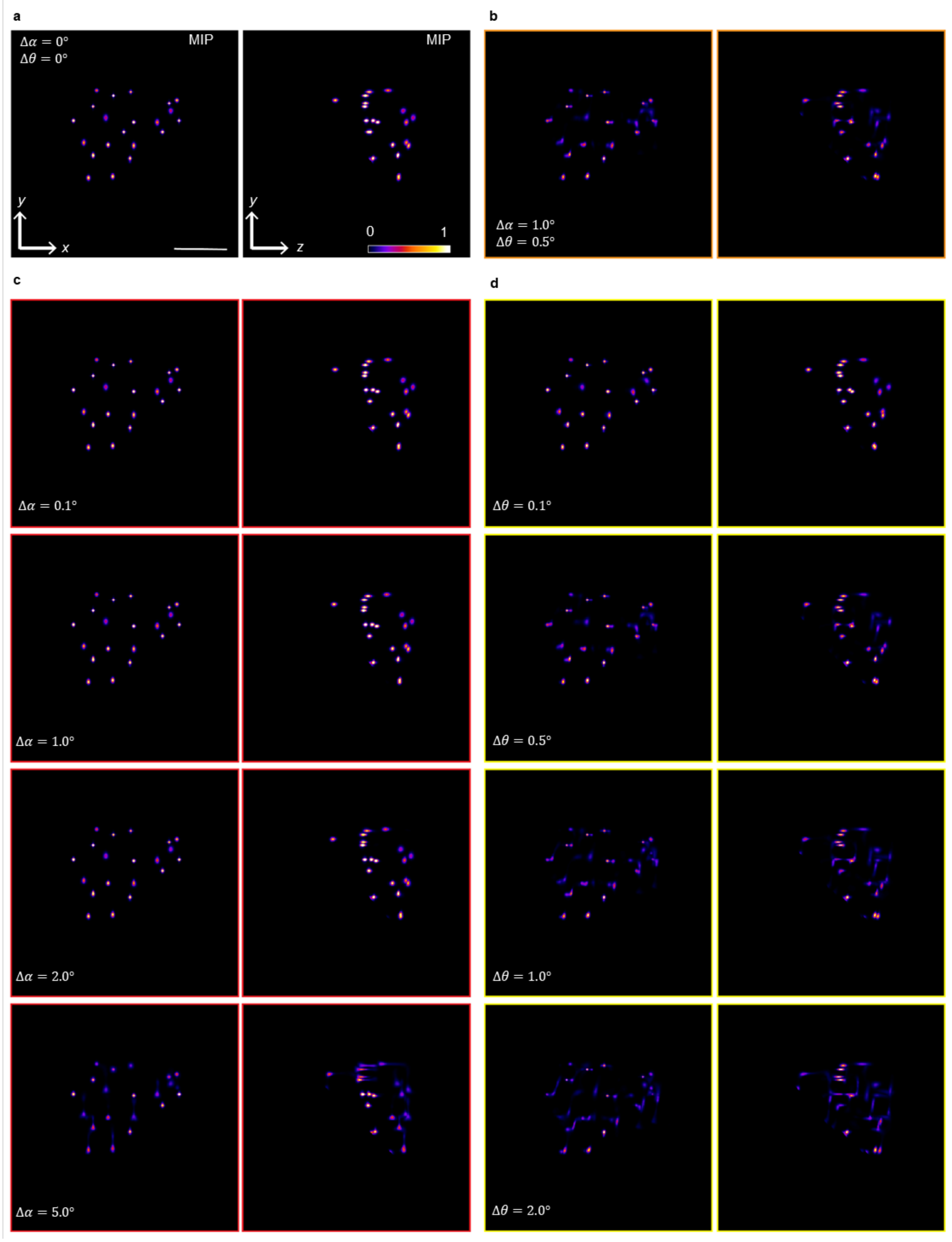

Comparisons of MiSLFM reconstructions with different levels of error for the estimations of tilted angle α and rotated angle θ. (a) Maximum intensity projections (MIP) on z directions and x direction of ground truth reconstruction for simulated 3D sample with randomly distributed beads under correct estimation of the mirror angle ($\alpha = 45°, \theta = 0°$). (b) Corresponding MIPs with inaccurate estimations on both angles in the reconstruction process

with same beads distribution of (a). The results have shown obvious artifacts on both side projections. (c) Comparisons on different levels of inaccurate estimations on the tilted angle α, demonstrating that obvious reconstruction artifacts appear starting from $\Delta\alpha > 2°$. (d) Comparisons on different levels of inaccurate estimations on the rotated angle θ. Compared to α, the accuracy of the rotated angle is more sensitive to reconstruction. Obvious reconstruction artifacts appear starting from $\Delta\theta > 0.5°$. Scale bar is 10 µm.

Supplementary Figure 9

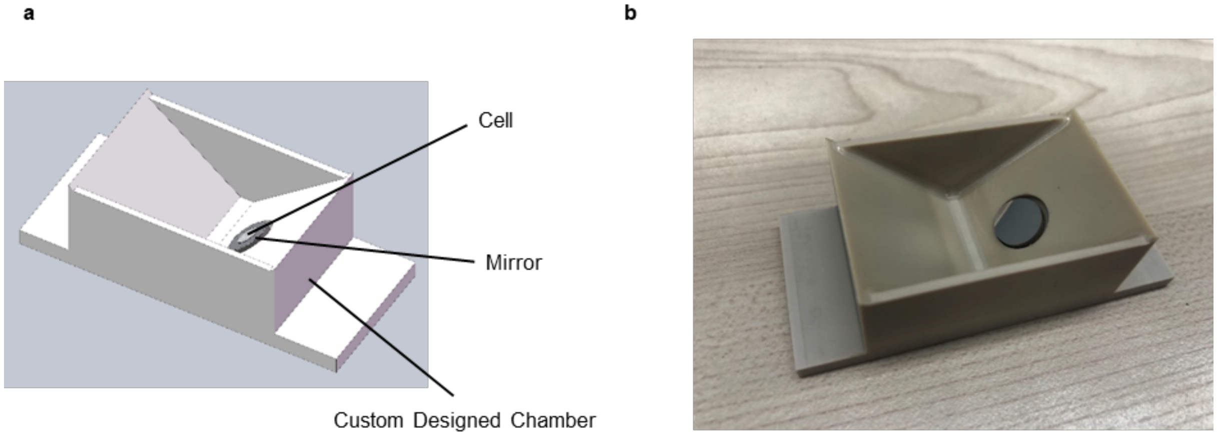

The structure of sample chamber and mounting.
(a) A diagram of the specimen and chamber, in which cells are seeded on top of a 25-nm SiO_2 coated first-surface mirror and the mirror is mounted on a custom-designed chamber. (b) A photogram of the SiO_2 coated mirror mounted on the custom designed chamber

Supplementary Figure 10

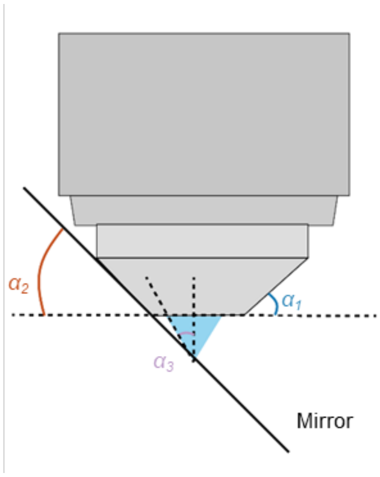

Schematic diagram of angles for objective (α_1), sample chamber (α_2) and effective NA (α_3). The selected objective and designed chamber can make these angles satisfy that $\alpha_1 + \alpha_3 \leq 90°, \alpha_2 \leq \alpha_1$. Therefore, the effective NA of objective will not be decreased.

Supplementary Table 1

Optical objective and illumination density in experiments

**Figure**	**Objective**	**Input illumination density**
Figure 1b & Supplementary Figure 1	Olympus 40×, NA 0.8 water immersive objective (LUMPLFLN40XW)	640nm laser, 3.01 mW mm^{-2}
Figure 2	Olympus 40×, NA 0.8 water immersive objective (LUMPLFLN40XW)	488/561 nm laser, 3.95 mW mm^{-2}(488 nm), 1.99 mW mm^{-2}(561 nm)
Figure 3	Olympus 40×, NA 0.8 water immersive objective (LUMPLFLN40XW)	488 nm laser, 0.22 mW mm^{-2}
Figure 4	Olympus 60×, NA 1.1 water immersive objective (LUMFLN60XW)	488/561 nm laser, 1.71 mW mm^{-2}(488 nm), 1.54 mW mm^{-2}(561 nm)
Figure 5 & Supplementary Figure 5	Olympus 20×, NA 0.5 water immersive objective (UMPLFLN20XW)	Mercury lamp, 488/561 nm 0.54 mW mm^{-2}(488 nm), 0.71 mW mm^{-2}(561 nm)
Supplementary Figures 2	Olympus 40×, NA 0.8 water immersive objective (LUMPLFLN40XW)	Mercury lamp, 488 nm 4.34 mW mm^{-2}
Supplementary Figure 3	Olympus 40×, NA 0.8 water immersive objective (LUMPLFLN40XW)	Simulation
Supplementary Figure 4	Nikon 40×, NA 0.95 objective (CFI Plan Apochromat Lambda 40XC)	488 nm laser, 0.44 mW mm^{-2}
Supplementary Figure 8	Olympus 40×, NA 0.8 water immersive objective (LUMPLFLN40XW)	Simulation

Supplementary Note 1: Reconstruction Pipeline

We designed a mirror enhanced reconstruction algorithm based on sLFM to independently reconstruct each frame of MiSLFM data into an isotropic 3D volume. Below we provide a detailed protocol of the reconstruction pipeline of MiSLFM. In addition, an overview of the entire process is provided in Fig. S1.

PSF generation

In order to get rid of the influence of system aberration, we combine the real capture fluorescent beads image (single slice) and the simulated PSF to obtain a PSF that is closer to the real situation.

First, take a single-layer fluorescent beads image as a reference for the real system PSF. Subsequently, by estimating the difference between the simulated PSF and the single-layer beads image, the system aberration is estimated. Finally, by using the calculated aberration distribution, the full-depth simulated PSF is calculated as the real system PSF for volume reconstruction. The simulated PSF parameters will affect some properties of the reconstructed volume, such as depth and reconstruction density. For the 40×/0.8 NA objective, our simulated PSF uses the following parameters: magnification 46, NA 0.8, MLpitch 97.5 µm, n 1.33, fml 1950µm, wavelength 525nm, OSR 3, z-spacing 0.14 µm, zmin -35 µm, zmax 35 µm, Nnum 15. For the 20×/0.5 NA objective: magnification 23, NA 0.5, MLpitch 97.5 µm, n 1.33, fml 1950 µm, wavelength 525 nm, OSR 3, z-spacing 0.56 µm, zmin -141 µm, zmax 141 µm, Nnum 15. For the 60×/1.1 NA objective: magnification 69, NA 1.1, MLpitch 97.5 µm, n 1.33, fml 1950 µm, wavelength 525 nm, OSR 3, z-spacing 94 nm, zmin -19 µm, zmax 19 µm, Nnum 15.

SLFM deconvolution

After PSF generation, the original data will be realigned and the region of interest will be selected at the same time. The selected area will then go through the sLFM phase space reconstruction, which uses the algorithm from[1], as shown in the following formula:

$$g_{j+1}^{k}(x,z) \leftarrow w_{u_j} g_j^k(x,z) \odot BP\left(M(x,u_j)\right) \Big/ \left(BP\left(FP\left(g_j^k(x,z)\right)\right)\right) + \left(1 - w_{u_j}\right) g_j^k(x,z) \qquad (S1)$$

Where $g_j^k(x,z)$ is the reconstructed volume in every full iteration k and frequency iteration u_j , w_{u_j} is a weight used to balance the different shot noise for different spatial frequency components, $\odot$ represents the dot product process, $BP(\cdot)$ represents the backpropagation process, $FP(\cdot)$ represents the front propagation process and $M(x,u_j)$ is the realigned measurement for the captured data.

Mirror modeling

To model the mirror position, we used the following equation to describe the position of the mirror:

$$P(a_1,a_2,a_3,a_4) \Longrightarrow a_1 + a_2x + a_3y + a_4z = 0 \qquad (S2)$$

However, since the mirror surface is never perpendicular to the x-y plane, $a_4 \neq 0$, let:

$$b_1 = -\frac{a_1}{a_4}, b_2 = -\frac{a_2}{a_4}, b_3 = -\frac{a_3}{a_4} \qquad (S3)$$

In this way, the expression of the plane can be transformed into:

$$P(b_1,b_2,b_3) \Longrightarrow z = b_1 + b_2x + b_3y \qquad (S4)$$

Where $\boldsymbol{b} = \begin{bmatrix} b_1 \\ b_2 \\ b_3 \end{bmatrix}$ are the parameters used to describe the mirror plane in 3D.

Volume warping

Since we need to perform the operations of mirror symmetry on the volume by multiple times, we usually first generate a mirror-symmetry matrix based on the position of the mirror, and pre-calculate the position of each voxel after symmetry, which is convenient for quick interpolation indexing. For the normal vector of the mirror $\boldsymbol{n} = \begin{bmatrix} b_2 \\ b_3 \\ -1 \end{bmatrix}$ and two symmetry points $\boldsymbol{C} = \begin{bmatrix} x_C \\ y_C \\ z_C \end{bmatrix}$, $\boldsymbol{D} = \begin{bmatrix} x_D \\ y_D \\ z_D \end{bmatrix}$, we have the following equation:

$$\begin{cases} \boldsymbol{C} - \boldsymbol{D} = k\boldsymbol{n} \\ \boldsymbol{n}^T \left(\dfrac{\boldsymbol{C} + \boldsymbol{D}}{2} \right) + b_1 = 0 \end{cases} \quad (S5)$$

where k is a non-zero value, $\boldsymbol{n}^T$ is the transform of normal vector $\boldsymbol{n}$. Based on this equation, we can solve D as:

$$\boldsymbol{D} = \boldsymbol{C} - 2\frac{\boldsymbol{n}^T\boldsymbol{C} + b_1}{\boldsymbol{n}^T\boldsymbol{n}}\boldsymbol{n} \quad (S6)$$

Then, we calculate the position of each discrete point in the entire volume after mirror symmetry, and save this mapping matrix W_P to facilitate the subsequent calculation of mirror estimation and volume reconstruction as below:

$$W_P(\boldsymbol{V}) = \boldsymbol{V} - 2\frac{\boldsymbol{n}^T\boldsymbol{V} + b_1}{\boldsymbol{n}^T\boldsymbol{n}}\boldsymbol{n} \quad (S7)$$

where $\boldsymbol{V} \in \mathbb{R}^{3\times N}$ is voxel-wised for each 3D vector of position in the reconstruction volume, N is the number of voxels. It should be noted that, whenever the mirror position changes, the corresponding mapping matrix need to be calculated again.

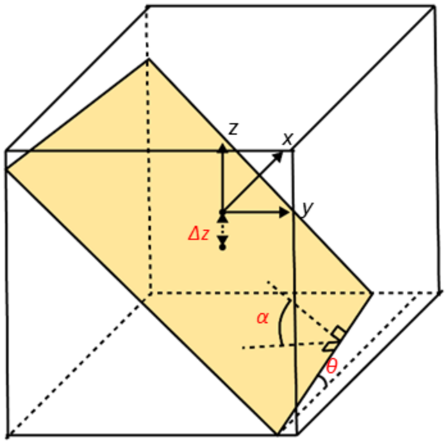

Fig. SN1.1 Schematic diagram to model the mirror position for MiSLFM. The mirror's position in the reconstructed volume can be represented by 3 independent variables: the vertical distance between the center of the reconstructed volume and its projection point along z-axis on the mirror Δz, the angle between the mirror plane and the horizontal plane α, and the rotation angle θ of the mirror around the z-axis.

Mirror estimation

The position of the mirror is affected by three variables: the vertical distance between the center of the reconstructed volume and its projection point along z-axis on the mirror Δz, the angle between the mirror plane and the horizontal plane α, and the rotation angle of the mirror around the z-axis θ (Fig. SN1.1).

However, since the holder with the mirror surface is made in advance, its angle is determined during the selection of the objective. We usually consider the angle between the mirror surface and the plane α to be a fixed value, which is measured in advance. In this case, the problem we need to solve degenerates from the usual three-variable optimization problem to a two-variable problem. Base on the normal vectors $\boldsymbol{n} = \begin{bmatrix} b_2 \\ b_3 \\ -1 \end{bmatrix}$ and $\boldsymbol{n_0} = \begin{bmatrix} 0 \\ 0 \\ 1 \end{bmatrix}$ of mirror surface plane and horizontal plane, we have:

$$cos(\alpha) = \frac{\boldsymbol{n}'\boldsymbol{n_0}}{|\boldsymbol{n}| \times |\boldsymbol{n_0}|} \tag{S8}$$

From this, we have

$$b_3 = \pm\sqrt{\frac{1}{cos^2 \alpha} - b_2^2 - 1} \tag{S9}$$

Base on the actual situation, we usually take the negative sign:

$$b_3 = F(b_2) = -\sqrt{\frac{1}{cos^2 \alpha} - b_2^2 - 1} = -\sqrt{tan^2 \alpha - b_2^2} \tag{S10}$$

Therefore, the equation of the plane is further rewritten as:

$$P(b_1, b_2) \Longrightarrow z = b_1 + b_2 x + F(b_2) y \tag{S11}$$

Similarly, we can also have

$$\begin{gathered} \Delta z = b_1 \\ cos\theta = \frac{-b_3}{\sqrt{\frac{1}{cos^2 \alpha} - 1}} = \frac{\sqrt{tan^2 \alpha - b_2^2}}{tan(\alpha)} \end{gathered} \tag{S12}$$

In the optimization process, to ensure the optimization effect, the value of b_2 is limited in a small range near zero. Because the rotation angle θ is usually a quite small value.

Finally, we use the preliminary reconstruction volume $\boldsymbol{V}$ from the sLFM and the mapping matrix W_P based on the mirror position $P(b_1, b_2)$ to optimize the parameters of the plane $\boldsymbol{b} = \begin{bmatrix} b_1 \\ b_2 \\ F(b_2) \end{bmatrix}$ as follows:

$$P^*(b_1, b_2) = \underset{P}{\operatorname{argmin}}\left(-\boldsymbol{V} \odot W_P(\boldsymbol{V})\right) = \underset{P}{\operatorname{argmin}}\left(-\boldsymbol{V} \odot \left(\boldsymbol{V} - 2\frac{\boldsymbol{n}^T\boldsymbol{V} + b_1}{\boldsymbol{n}^T\boldsymbol{n}}\boldsymbol{n}\right)\right) \tag{S13}$$

where ⨀ represents the voxel-wise dot-product process. We use the *fminsearch* function in the MATLAB to search the optimal point. When the estimated value of θ is within 0.5° of the accurate value, it will not affect the accuracy of the 3D reconstruction results (Fig. S8). When the particles in the adherent cells reconstructed by the sLFM method are highly overlapped with their own virtual images in the mirror,

the mirror position estimation will have a large deviation. But in this case, the sample is a thin 2D structure, which is usually unnecessary for 3D imaging. In most cases, the samples such as cells have certain thickness with structures not on the mirror plane. Our algorithm can provide an accurate estimation of the mirror position.

Mirror enhanced deconvolution

Once the mirror position $P^*(b_1, b_2)$ is determined, we calculate the final warping matrix W_{P^*} for the data and conduct the deconvolution with a two-step deconvolution in each iteration. In sLFM, each phase-space measurement will be independently input into the reconstruction algorithm to optimize the entire volume, which is described as:

$$g_{j'}^{k}(x,z) \leftarrow D_{SLFM}\left(M(x,u_j), g_j^k(x,z)\right) \tag{S14}$$

Furthermore, for each input angle, we added a symmetric mapping deconvolution process. Since the measurement comes from the sum of two symmetrical volumes, the symmetrical volume should also satisfy the light-field imaging model. Based on this, we perform the second deconvolution as follows:

$$g_{j+1}^{k}(x,z) \leftarrow W_{P^*}\left(D_{SLFM}\left(M(x,u_j), W_{P^*}\left(g_{j'}^{k}(x,z)\right)\right)\right) \tag{S15}$$

We first warp the volume $g_{j'}^{k}(x,z)$ by the symmetrical mapping W_{P^*}. Then a similar deconvolution reconstruction process is performed. Later, we re-warp the volume back to the original perspective. Finally, by using the mirror parameters, the mirror enhanced reconstruction result is cropped to remove the symmetry volume in the results.

An overview of the entire process is provided in Fig. S7**.**

Supplementary Note 2: Artifacts of MiSLFM

In sLFM, the PSF gradually deteriorates as it moves away from the focal plane along the z-axis, which means the kernel size of PSF is gradual increases (Fig. SN2.1a). In the mirror view, the resolution decreasing axis changes from axial to lateral in mirror view (Fig. SN2.1b), with this effect, the mixture view has been greatly improved by maintaining the better resolution of the two views, therefore an approximately isotropic PSF is obtained (Fig. SN2.1c). Meanwhile, the PSF with a mixture view would be non-uniformed in space. The size of the PSF is not only related to the axial distance to the mirror, but also related to the lateral distance to the mirror (Fig. 5a, Fig. 6b). Furthermore, since the resolution of the mixture view is affected by the two views, the accuracy of the mirror estimation will also affect the isotropic performance of the resolution. In addition, different angles of mirror setups also affect the resolution performance (Fig. 6).

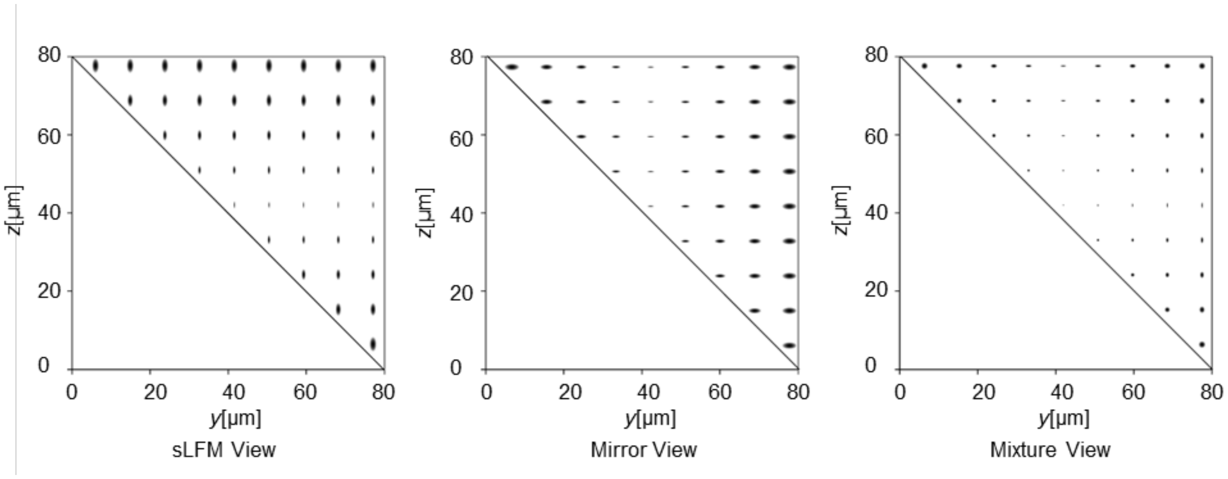

Fig. SN2.1 Schematic diagram of resolution artifacts under MiSLFM reconstruction. These 3 views show the resolution decreasing along with different directions, and their mixture effect on the mixture view. (a) PSF distribution of sLFM. (b) PSF distribution of mirror view of sLFM. (c) The mixture view keeps the best resolution on both sLFM view and mirror view, therefore, it achieves an isotropic PSF. However, the size of the mixture view PSF varies in both Z and Y directions, which makes it a non-uniform PSF in neither Z or Y direction, but it still improves the axial resolution, compared to sLFM. Note that the size of the PSFs is for visualization purposes only.

Supplementary References:

1. Lu, Z. et al. Phase-space deconvolution for light field microscopy. Opt Express 27, 18131-18145, doi:10.1364/oe.27.018131 (2019).
